# Postoperative Morbidity and Complications in Elderly Patients after Harvesting of Iliac Crest Bone Grafts

**DOI:** 10.3390/medicina57080759

**Published:** 2021-07-27

**Authors:** Marie Sophie Katz, Mark Ooms, Marius Heitzer, Florian Peters, Philipp Winnand, Kristian Kniha, Stephan Christian Möhlhenrich, Frank Hölzle, Matthias Knobe, Ali Modabber

**Affiliations:** 1 Department of Oral and Maxillofacial Surgery, University Hospital RWTH Aachen, Pauwelsstraße 30, 52074 Aachen, Germany; mooms@ukaachen.de (M.O.); mheitzer@ukaachen.de (M.H.); flpeters@ukaachen.de (F.P.); pwinnand@ukaachen.de (P.W.); kkniha@ukaachen.de (K.K.); fhoelzle@ukaachen.de (F.H.); amodabber@ukaachen.de (A.M.); 2 Department of Orthodontics, University Witten/Herdecke, Alfred-Herrhausen-Straße 45, 58448 Witten, Germany; stephan.moehlhenrich@uni-wh.de; 3 Department of Trauma and Reconstructive Surgery, University Hospital RWTH Aachen, Pauwelsstraße 30, 52074 Aachen, Germany; matthias.knobe@luks.ch; 4 Department of Orthopaedic and Trauma Surgery, Lucerne Cantonal Hospital, 6000 Lucerne, Switzerland

**Keywords:** elderly patients, iliac crest bone graft, oral augmentation, postoperative morbidity, gait disturbances, prolonged hospital stay

## Abstract

*Background and objectives*: In oral and maxillofacial operations, the iliac crest is a commonly used donor site from which to harvest bone for augmentation prior to dental implantation or for reconstruction of jaw defects caused by trauma or pathological lesions. In an aging society, the proportion of elderly patients undergoing iliac crest bone grafting for oral augmentation is growing. Although postoperative morbidity is usually moderate to low, the age and health of the patient should be considered as risk factors for complications and delayed mobilization after the operation. The aim of this retrospective study was to evaluate the postoperative morbidity and complications in elderly patients after the harvesting of iliac crest bone grafts for oral surgery. *Material and Methods*: Data were collected from a total of 486 patients (aged 7–85) who had a surgical procedure that included the harvesting of iliac crest bone grafts for intraoral transplantation. All patients were operated on between 2005 and 2021 in the Department for Oral and Maxillofacial Surgery of the University Hospital in Aachen, Germany. As parameters for postoperative morbidity and complications, gait disturbances, hypesthesia of cutaneous nerves, incision hernias, iliac crest fractures, delayed wound healing, and unfavorable scar formation at the donor site were all evaluated. *Results*: The study was performed with 485 patients due to the exclusion of one patient as the only one from whom grafts were taken from both sides. When younger and older patients were compared, neither gait disturbances (*p* = 0.420), nor hernias (*p* = 0.239), nor fractures (*p* = 0.239), nor hypesthesia (*p* = 0.297), nor wound healing delay (*p* = 0.294), nor scar problems (*p* = 0.586) were significantly different. However, the volume of the graft was significantly correlated with the duration of the hospital stay (ρ = 0.30; *p* < 0.01) but not with gait disturbances (ρ = 0.60; *p* = 0.597). Additionally, when controlling for age (*p* = 0.841), sex (*p* = 0.031), ASA class (*p* = 0.699), preexisting orthopedic handicaps (*p* = 0.9828), and the volume of the bone graft (*p* = 0.770), only male sex was associated with the likelihood of suffering gait disturbances (*p* = 0.031). *Conclusions*: In conclusion, harvesting bone grafts from the anterior iliac crest for intraoral augmentation is a safe procedure for both young and elderly patients. Although there is some postoperative morbidity, such as gait disturbances, hypesthesia, scar formation, or delayed wound healing at the donor site, rates for these minor complications are low and mostly of short duration. Major complications, such as fractures or incision hernias, are very rare. However, in our study, the volume of the bone graft was associated with a longer stay in hospital, and this should be considered in the planning of iliac crest bone graft procedures.

## 1. Introduction

In oral and maxillofacial operations, the iliac crest is a commonly used donor site from which to harvest bone for augmentation prior to dental implantation or for reconstruction of jaw defects caused by trauma or pathological lesions [1,2,3]. Progredient bone resorption after tooth extraction often requires additional bone augmentation procedures before the placement of dental implants [4]. Especially in cases of extended atrophy, vertical augmentations can be a challenging initial situation, and there are different techniques like sandwich-, onlay-, and inlay-osteoplasty to place the bone graft in an optimal way [5,6]. Osseointegration has been defined as a direct and functional connection between bone and an artificial implant; both bone quality and quantity could influence the success of these procedures [7]. Due to its osteoconductive, osteoinductive, and osteogenetic characteristics, autologous bone is still used as the gold standard material in oral augmentation and is preferred over alloplastic bone substitutes [8,9,10]. Furthermore, bone blocks seem to maintain the volume of the initial augmentation site more than guided bone-regeneration techniques [11]. When the amount of bone that can be harvested intraorally is insufficient, the iliac crest is often the favored donor site compared to calvaria or tibia bone grafts [12,13,14]. Usually, bone grafts for oral augmentation are harvested from the anterior part of the iliac crest, where up to 26.29 mL of uncompressed cortico-cancellous graft can be gained [15]. If more bone is needed, as in extensive defects or augmentation of two atrophic jaws, bone grafts can also be harvested from the posterior iliac crest [15], which is associated with fewer complications but requires the repositioning of the patient and therefore extends the operating time [16,17].

Complications and postoperative morbidity after harvesting of anterior iliac crest bone grafts are generally low [18], but there is a certain percentage of pain, gait disturbance, superficial skin sensitivity disorders, and delayed wound healing at the donor site [17,19]. Nevertheless, major complications, such as fractures or hernias, are rare but have been described in case reports and reviews [20,21,22].

In an aging society, the proportion of elderly patients undergoing iliac crest bone grafting for oral augmentation is growing. Although postoperative morbidity is usually moderate to low in both children and adults [2,18,23,24], the age and health condition of the patient should be considered as risk factors for complications and delayed mobilization after the operation [20,21,25,26].

Although there are a few studies and reviews about complications after the harvesting of bone grafts from the anterior iliac crest [1,2,17,18,19,21,23,25,27], it remains unclear whether age and health status are significant risk factors for increased complications and greater morbidity or whether postoperative disturbances are mainly associated with the surgical approach and are irrespective of physical fitness.

The aim of this retrospective study was to evaluate postoperative morbidity and complications in elderly patients after harvesting iliac crest bone grafts for oral surgery.

## 2. Materials and Methods

The study was approved by the local Clinical Research Ethics Committee (Decision Number 211-21, 17 June 2021). 

This is a retrospective clinical study to analyze the postoperative morbidity and complications after harvesting of iliac crest bone grafts for intraoral use in patients aged 60 or older in comparison to younger patients.

### 2.1. Data Collection

Data were collected for patients who had a surgical procedure that included the harvesting of iliac crest bone grafts for intraoral transplantation between 2005 and 2021 in the Department for Oral and Maxillofacial Surgery of the University Hospital in Aachen, Germany.

Exclusion criteria were having an orthopedic operation during the same procedure or within one month after the procedure and patients who attended another clinic for their postoperative follow-ups. In order to have a comparable and homogenous study population, the one patient with two donor sites was excluded as well as all patients who had less than one month of clinical follow-up.

A total of 485 patients were identified for the study; of these, 116 patients were 60 or older at the date of their operation, and 369 patients were younger than 60.

**Figure 1:**
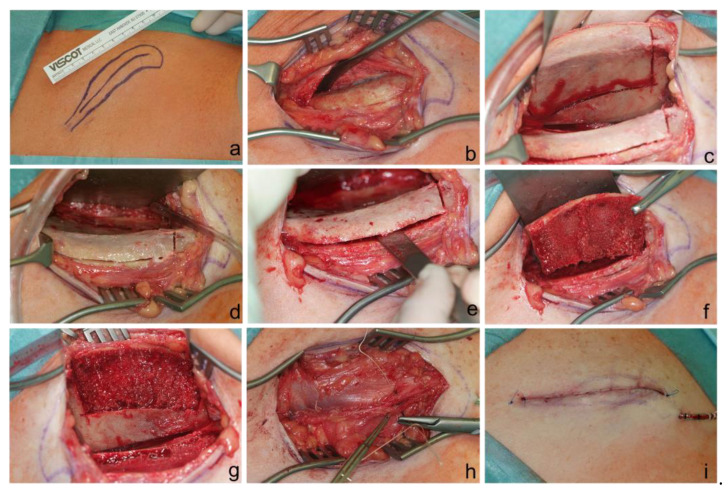
Procedure of grafting a cortical bone block from the right anterior iliac crest. (**a**) Marking of the anterior iliac crest. (**b**) Preparation of the anterior iliac crest. (**c**,**d**) Circumscribing the bone graft with a saw ((**c**): mirror instrument used). (**e**) Careful carving of the bone block. (**f**,**g**) Removal of the cortical block ((**g**): mirror instrument mirror used). (**h**) Iterative wound closure. (**i**) Closed wound with inserted drain.

In all patients, the grafts were taken from the anterior iliac crest, whereas in 203 cases, the graft was taken from the left side; in 282 cases, from the right side; and in one case, from both sides, as more bone was needed (Figure 1). 

The following indications were included: 185 patients underwent preimplantologic augmentation of an atrophic jaw. Of these, 102 received augmentation of the upper jaw and 60 of the lower jaw, while 23 patients had both mandible and maxilla augmentation during the same procedure. Another 87 patients had a cystic or traumatic defect in the upper jaw, while 175 patients had similar defects in the lower jaw, and 3 patients had defects in both upper and lower jaw. An additional 35 patients with a history of a cleft jaw and a residual bone defect underwent secondary or tertiary osteoplastic surgery.

In 97 patients, a cancellous bone graft was extracted; in 387 patients, a cortico-cancelllous bone block was sewed and chased, of which 376 cases were monocortical, and 11 cases were bicortical bone grafts.

After collecting the data from the medical records and anesthetic journals of the patients, we evaluated their health status at the time of the operation: 219 patients were assigned to ASA I, 208 into ASA II, and 58 were assigned to ASAIII. Furthermore, we divided patients by the type of their medical condition: 125 patients suffered from cardiovascular problems, 47 had a neurological or psychiatric illness in their history, 81 had metabolic diseases, and 20 had underlying orthopedic conditions, such as osteoporosis or arthrosis.

As parameters for postoperative morbidity and complications, we evaluated gait disturbances, hypesthesia of superficial cutaneous nerves, incision hernias, iliac crest fractures, delayed wound healing, and unfavorable scar formation.

### 2.2. Statistical Analysis

Differences between groups were analyzed by the chi-square test for categorical data (ASA classification, health condition, indication for the procedure, type of graft) and by the Mann–Whitney test for metrical data (graft volume, duration of postoperative hospital stay). Associations between parameters were analyzed by using the Spearman correlation (graft volume with hospital stay or gait disturbances). Logistic regression analysis was performed to compare age groups for gait disturbances, adjusted for sex, ASA class, orthopedic handicaps, and volume of bone graft. *p*-values < 0.05 were considered to be significant. 

The data were analyzed using the statistical software SPSS Statistics (Version 27, IBM Corp., Armonk, NY, USA) and GraphPad Prism (Version 6, GraphPad Software Inc., La Jolla, CA, USA). 

## 3. Results

The study was performed with 485 patients in total. The mean age of the patients was 43.92 years (SD ± 18.99). The patients were divided into two groups: younger patients (<60, group I) and elderly patients (≥60 years, group II).

Patients in group II were significantly more often in ASA class II or III (*p* < 0.01), they suffered more often from cardiovascular diseases (*p* < 0.01), from endocrine and metabolic diseases (*p* < 0.01), and of orthopedic handicaps (*p* < 0.01), whereas neuro-psychiatric illness was not significantly different between the two age groups (*p* = 0.527).

Elderly patients had significantly more frequent procedures for preimplantologic augmentation than younger patients (*p* < 0.01), and the filling of cystic or traumatic defects was not significantly different between the two groups (*p* = 0.172), while osteoplasty of residual cleft defects was, as to be expected, significantly more frequent in the younger patients (*p* < 0.01). Consistent with those figures, patients aged 60 or older needed cortical grafts significantly more often than cancellous grafts relative to the younger patients (*p* < 0.01) (Table 1).

**Figure 2:**
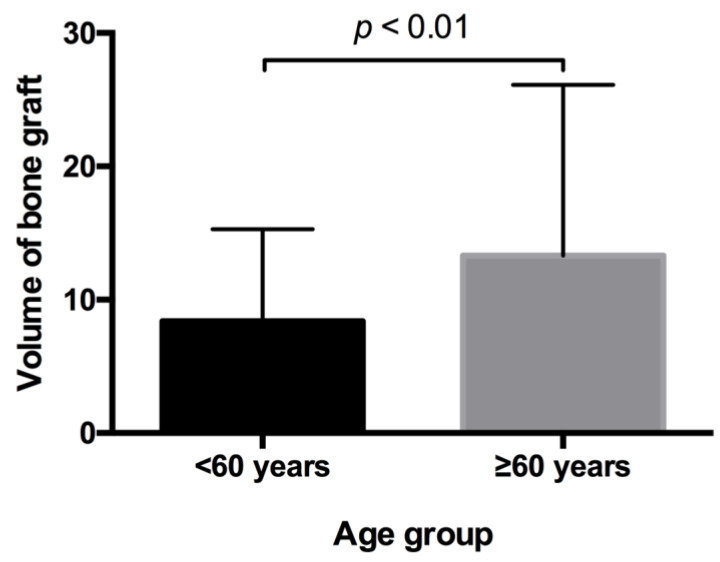
Comparison of groups for bone graft volume. The volume of the graft was documented in 315 out of 485 cases. In these, the mean bone graft was 8.39 cm^3^ (SD ± 6.91; Median 6 cm^3^) in younger patients and 13.32 cm^3^ (SD ± 12.787; Median 10 cm^3^) in elderly patients. *p*-value corresponding to testing for differences between groups with Mann–Whitney test (*p* < 0.01).

The volume of the graft was documented in 315 out of 485 cases. In these, the mean bone graft was 8.39 cm^3^ (SD ± 6.91; Median 6 cm^3^) in the younger patients and 13.32 cm^3^ (SD ± 12.787; Median 10 cm^3^) in the elderly patients, which was a significant difference (*p* < 0.01) (Figure 2). 

**Figure 3:**
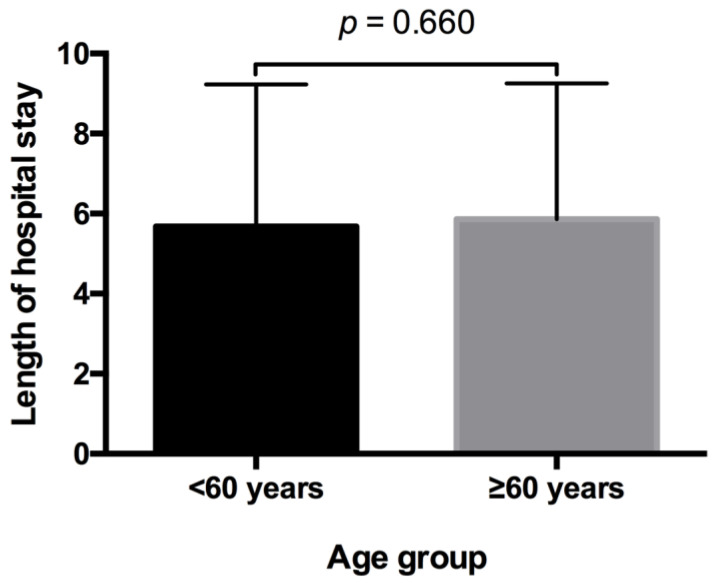
Comparison of groups for postoperative length of stay. The mean duration of stay after the procedure was 5.00 days for both groups (SD ± 3.55 in patients < 60 years; SD ± 3.40 in patients ≥ 60 years). *p*-value corresponding to testing for differences between groups with Mann–Whitney test (*p* = 0.660).

No significant difference was evident when comparing the duration of hospital stay of elderly patients with that of younger patients (*p* = 0.660). The mean duration of stay after the procedure was 5.00 days for both groups (SD ± 3.55 in patients < 60 years; SD ± 3.40 in patients ≥ 60 years) (Figure 3).

As parameters for postoperative morbidity and complications, we evaluated gait disturbances, hypesthesia of cutaneous nerves, incision hernias, iliac crest fractures, delayed wound healing, and unfavorable scar formation. In total, there was one fracture of the anterior iliac crest without dislocation in a 76-year-old woman, which was treated conservatively, and one case of an incision hernia in a 70-year-old male patient. Of the 485 patients, 74 complained of gait disturbances, 10 had documented superficial hypesthesia, 14 patients were affected by delayed wound healing, and 6 patients by unfavorable scar formation at the donor site.

Neither gait disturbances (*p* = 0.420), nor hernias (*p* = 0.239), nor fractures (*p* = 0.239), nor hypesthesia (*p* = 0.297), nor wound healing delay (*p* = 0.294), nor scar problems (*p* = 0.586) were significantly different in a comparison of the younger and older patients.

Furthermore, postoperative morbidity was not significantly affected by whether a cancellous or cortical graft was used (gait disturbances, *p* = 0.570; hernias, *p* = 1.000; fractures, *p* = 1.000; hypesthesia, *p* = 0.424; wound healing, *p* = 0.058; scar formation, *p* = 0.218).

However, the volume of the graft was significantly correlated with the duration of hospital stay (ρ = 0.30; *p* < 0.01) but not with gait disturbances (ρ = 0.60; *p* = 0.597). 

When controlling for age (*p* = 0.841), sex (*p* = 0.031), ASA class (*p* = 0.699), preexisting orthopedic handicaps (*p* = 0.9828), and the volume of the bone graft (*p* = 0.770), the only significant difference was that male patients suffered more gait disturbances than female patients (*p* = 0.031) (Table 2).

## 4. Discussion

The iliac crest is a commonly used donor site from which to harvest bone for augmentation of atrophic jaws or for filling intraoral defects after cyst removal or traumatic bone loss. However, this procedure is sometimes associated with postoperative discomfort and also rarely with major complications [1,2,17,18,19,20,21,22,23,25]. Although the proportion of elderly patients is growing, it remains unclear if the age is a risk factor for postoperative morbidity, prolonged hospital stays, or complications.

While the overall rate of complaints after iliac crest graft procedures is generally low [18,23,28], gait disturbances are the most common morbidity [29,30], and yet, they are rarely chronic and seldom last a long time [2,17]. In our findings, 74 out of 485 patients suffered from this condition (15.26%), but only 11 patients (0.23%) had chronic gait problems that lasted for more than a month, which is lower than that described by Brudnicki et al. [1] and Schaaf et al. [23] and similar to that described by Nkenke et al. [17]. We found no significant difference in gait disturbances between the younger patients and the patients aged 60 or older and no correlation with the volume of the graft. Moreover, in contrast to other studies, our analysis controlled for other patient- and graft-specific aspects so as to isolate their impact on gait disturbances, and only male sex was found to have a statistically significant effect on the incidence of gait problems. This is unlike the findings described by Schaaf et al. [23], but their study did not include regression analysis, so their findings may be influenced by the heterogeneity of their study sample and the volume of their grafts. An explanation for our finding could be that the men were more active after the procedure or else were more likely to express complaints, but since our data analysis was retrospective and there was no standardized postoperative questionnaire, our study findings are limited to the known health and procedure data.

Hypesthesia in the region of the donor site was found in 10 patients of our study population (2.06%). Only five of these (1.03%) had superficial sensory issues that lasted for more than a month after the operation, and there was no difference between the younger and older patients. This is in line with the findings of Nkenke et al. [17], who found a significant tendency towards sensorial recovery after one month, and it is similar to the study of Schaaf et al. in which 2.7% of patients suffered from persistent sensory disturbances [23].

We found delayed wound healing in 14 cases (2.89%) and unfavorable scar formation in 6 cases (1.24%), with no significant difference between the age groups. Here, our results were lower than those of Tosun et al., who detected hematoma in 8.1% and infection in 12.8% of cases [30], and also lower than those of Boehm et al., who described higher rates of hypertrophic and painful scars (9.1%) [29]. Therefore, the operation technique may have a greater impact on scar formation than age and its associated reduced skin elasticity.

Concerning major complications, such as anterior iliac crest fracture and incision hernias, our study included one case of each (0.21% for fractures and 0.21% for hernia), indicating that this is rare and in line with case reports from Ovalioglu et al. [27], Gawhale et al. [20], and Covani et al. [21], and a review by Nocini et al. [22]. Although both these patients were aged older than 60, there was no significant difference, as such major complications are very rare. In addition, as the case of fracture reported by Covani et al. was in a 47-year-old anorexic woman, this incidence may be associated with factors other than age.

The mean volume of the bone grafts in our study was 8.39 cm^3^ (SD ± 6.91; Median 6 cm^3^) in the younger patients and 13.32 cm^3^ (SD ± 12.787; Median 10 cm^3^) in the older patients, both of which were less than the average amount of 15 cm^3^ (range 9.0–25.5 cm^3^), as described in the study by Kessler et al. [16]. We found no correlation between the volume of the graft and complications even when controlling for the other variables. However, Kessler et al. described complications that were limited to the cases in which the volume of bone graft exceeded 17 cm^3^. Similarly, Ghassemi et al. described gait disturbances increasing in relation to the volume of bone harvested from the iliac crest [31]. It is understandable that complications increase in relation to the amount of bone harvested due to anatomical and stability reasons, but when considering the size of the population of patients in our study (485), it suggests that, in most situations, a smaller amount of harvested bone is needed, and complications are tradeable for bone volume. 

Last but not least, we found an association between the volume of the graft and the length of the postoperative hospital stay, which underlines that, even though we could not detect significantly more complications or gait disturbances, the size of the graft is likely to have an impact on the rehabilitation of physical fitness. New implant shapes and geometries might also reduce the need for extensive bone augmentations and may therefore indirectly decrease the complications and the hospital stay associated with iliac crest bone grafts [32,33].

Furthermore, although the elderly patients were generally unhealthier and more frequently in ASA class III than the patients aged younger than 60, our study found no association between age and longer hospital stays in our study.

Complementary to the findings of Brudnicki et al., who concluded that alveolar bone grafting at an earlier age does not increase donor site symptoms [1], our study states that elderly patients experience no increased complications or morbidity when bone is harvested the iliac crest for intraoral transplantation.

## 5. Conclusions

In conclusion, harvesting bone grafts from the anterior iliac crest for intraoral augmentation is a safe procedure for both young and elderly patients. Although there is some postoperative morbidity, such as gait disturbances, hypesthesia, scar formation, or delayed wound healing at the donor site, the incidence of these minor complications is low, and they are mostly of short duration. Major complications, such as fractures or incision hernias, are very rare. However, in our study, the volume of the bone graft was associated with a longer stay in hospital, which should be considered in the planning of iliac crest bone graft procedures. Further prospective clinical studies on this topic are needed.

## Figures and Tables

**Table 1:** Indications for iliac crest bone grafts.

Age Group	Augmentation	Filling of Traumatic/Cystic Defects	Osteoplasty of Residual Cleft Defects	Total
Group I (<60 years)	126	208	34	368
Group II (≥60 years)	59	57	1	117
Total	185	265	35	485

Elderly patients had significantly more frequent procedures for preimplantologic augmentation than younger patients (*p* < 0.01); the filling of cystic or traumatic defects was not significantly different between the two groups (*p* = 0.172), while osteoplasty of residual cleft defects was, as to be expected, significantly more frequent in the younger patients (*p* < 0.01).

**Table 2:** Regression analysis performed for evaluation of gait disturbances.

Parameter	Comparison	Odds Ratio	*p*-Value
Age	<60 vs. ≥60 years	1.078	0.841
Sex	Male vs. Female	0.489	**0.031**
ASA Class	Class I/II vs. Class III	1.189	0.699
Orthopedic handicaps	No vs. Yes	1.158	0.828
Volume of bone graft	cm^3^	0.994	0.770

When controlling for age, sex, ASA class, preexisting orthopedic handicaps, and the volume of the bone graft, the only significant difference was that male patients suffered more gait disturbances than female patients (*p* = 0.031).

## Data Availability

Data available on request due to privacy restrictions. The data presented in this study are available on request from the corresponding author. The data are not publicly available due to privacy restrictions.

## Abbreviations

ASA	American Society of Anesthesiologists
SD	Standard Deviation
